# Supplementary material for: Additive manufacturing of alloys with programmable microstructure and properties
Source: Nat Commun. 2023 Oct 30;14:6752. doi: 10.1038/s41467-023-42326-y (PMC10616214; doi:10.1038/s41467-023-42326-y)
Supplement: Supplementary file 1 — Supplementary Information [file 41467_2023_42326_MOESM1_ESM.pdf]

Supplementary materials

Additive manufacturing of alloys with programmable microstructure and properties

Shubo Gao[1,2], Zhi Li[3], Steven Van Petegem[4], Junyu Ge[1], Sneha Goel[4,5], Joseph Vimal Vas[6], Vladimir Luzin[7], Zhiheng Hu[2], Hang Li Seet[2], Dario Ferreira Sanchez[4], Helena Van Swygenhoven[4], Huajian Gao[1,3], Matteo Seita[8,*]

[1]School of Mechanical and Aerospace Engineering, Nanyang Technological University, Singapore 639798, Republic of Singapore

[2]Additive Manufacturing Division, Singapore Institute of Manufacturing Technology (SIMTech), Agency for Science, Technology and Research (A*STAR), Singapore 636732, Republic of Singapore

[3]Institute of High Performance Computing, Agency for Science, Technology and Research (A*STAR), Singapore 138632, Republic of Singapore

[4]Photon Science Division, Paul Scherrer Institute, Villigen 5232, Switzerland

[5]VTT Technical Research Centre of Finland, Espoo 02150, Finland.

[6]School of Materials Science and Engineering, Nanyang Technological University, Singapore 639798, Republic of Singapore

[7]Australian Nuclear Science & Technology Organisation (ANSTO), Lucas Heights, NSW 2234, Australia

[8]Department of Engineering, University of Cambridge, Cambridge CB2 1PZ, United Kingdom

*Corresponding author: Matteo Seita (ms2932@eng.cam.ac.uk)

This Supplementary Information (SI) contains five supplementary tables (Supplementary Tables 1–5) and ten supplementary figures (Supplementary Figs. S1–S10).

Supplementary Table 1. Tensile properties of layered microstructures produced and the respective single components. Each value was averaged by at least three tensile tests.

Sample	YS (MPa)	UTS (MPa)	Strain to UTS (%)	Elongation (%)
Non-recrystallized	439.6± 9.1	692.2±0.7	32.1±1.9	45.6±2.4
Recrystallized	322.2±10.0	673.9±3.3	45.8±2.1	62.2±1.2
Fine layered	395.9±12.7	708.4±3.9	38.9±0.4	54.4±1.0
Coarse layered	416.4±9.7	689.9±17.9	40.9±2.8	51.8±4.5

Supplementary Table 2. Chemical composition of the as-received SS316L powders measured by inductively coupled plasma (in wt.%).

Cr	Ni	Mo	Mn	Si	Cu
17.26±0.10	12.78±0.04	2.40±0.04	0.79±0.09	0.62±0.01	0.2±0.01
Al	C	P	S	Fe	
0.06±0.01	0.027	<0.01	<0.01	Bal.	

Supplementary Table 3. Details of process parameters using the custom-made LPBF printer.

	Laser power	Scanning speed	Layer thickness	Scan rotation	Hatch spacing	Remelting
Parameter H	60 W	600 mm/s	10 μm	90º	25 μm	No
Parameter L	60 W	600 mm/s	10 μm	90º	10 μm	Yes
Other prints	60 W	600 mm/s	10 μm	90º	10 to 35 μm	No

Supplementary Table 4. Material (SS316L) parameters[1, 2] used for finite element model (FEM) simulation of LPBF process.

Parameters	Values
Liquidus temperature	1437 ºC
Solidus temperature	1390 ºC
Temperature dependent conductivity	(9.248e-2+1.571e-4×T)×100 (mW mm^{-1} $ºC^{-1}$)
Temperature dependent specific heat	462+0.164×T (mJ ton^{-1} $ºC^{-1}$)
Latent heat	290e9 (mJ ton^{-1})
Density	7.2e-9 (ton mm^{-3})
Thermal expansion coefficient	1.34e-5 ($ºC^{-1}$)
Yield strength	300 (MPa)
Young's modulus	190 (GPa)
Poisson's ratio	0.3

Supplementary Table 5. Model parameters for non-recrystallized phase, recrystallized phase, and IZ.

Parameters	Non-recrystallized	Recrystallized	IZ
Characteristic strain rate (s^{-1}), Φ	0.001	0.001	0.001
Strain rate sensitivity, m	0.005	0.005	0.005
Initial flow stress (MPa), σ_0	439.6	322.2	322.2
Transient flow stress (MPa), Q	426.2	107.7	107.7
Transient hardening constant, k_1	5.44	6.82	6.82
Strain hardening rate (MPa), H	354.2	1045.2	1045.2
Hardening exponent, k_2	0.70	0.623	0.623
Intrinsic material length (μm), α	--	--	5.57
Build-up of back stress from strain gradient (MPa μm), β	--	--	8.96e4
Back stress increasing rate coefficient, c	--	--	1.03

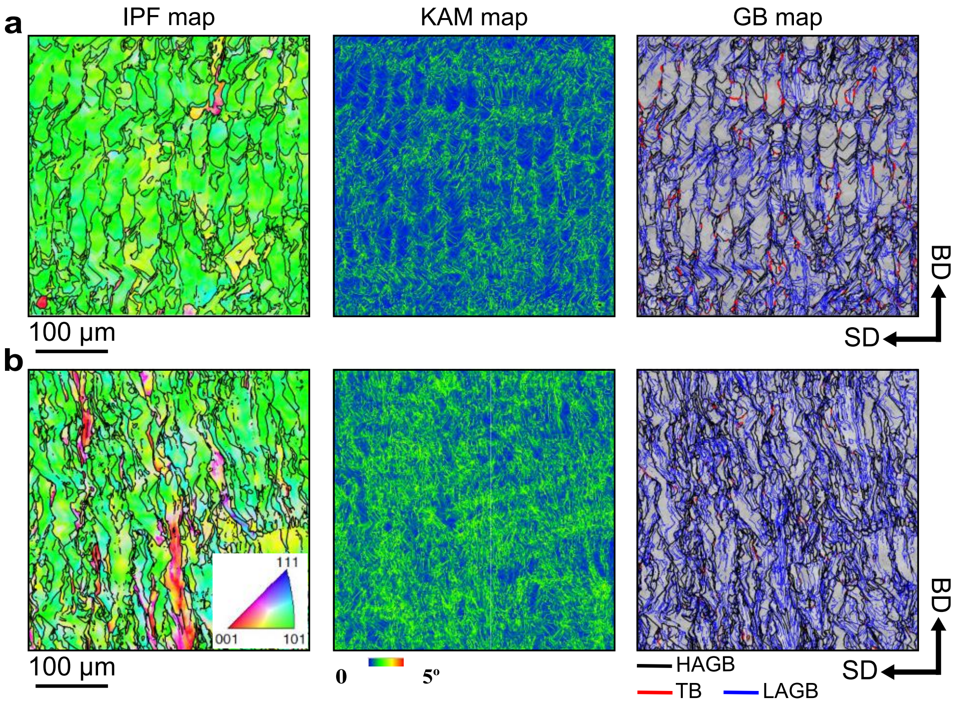

Supplementary Fig. 1. As-built microstructures of (a) H-SS316L and (b) L-SS316L. Electron backscatter diffraction (EBSD) measurements show crystal orientation (IPF, inverse pole figure) along the build direction, kernel average misorientation (KAM), and grain boundary (GB) character distribution. HAGB, LAGB, and TB represent high angle grain boundaries, low angle grain boundaries, and twin boundaries, respectively. BD and SD represent build direction and scanning direction, respectively.

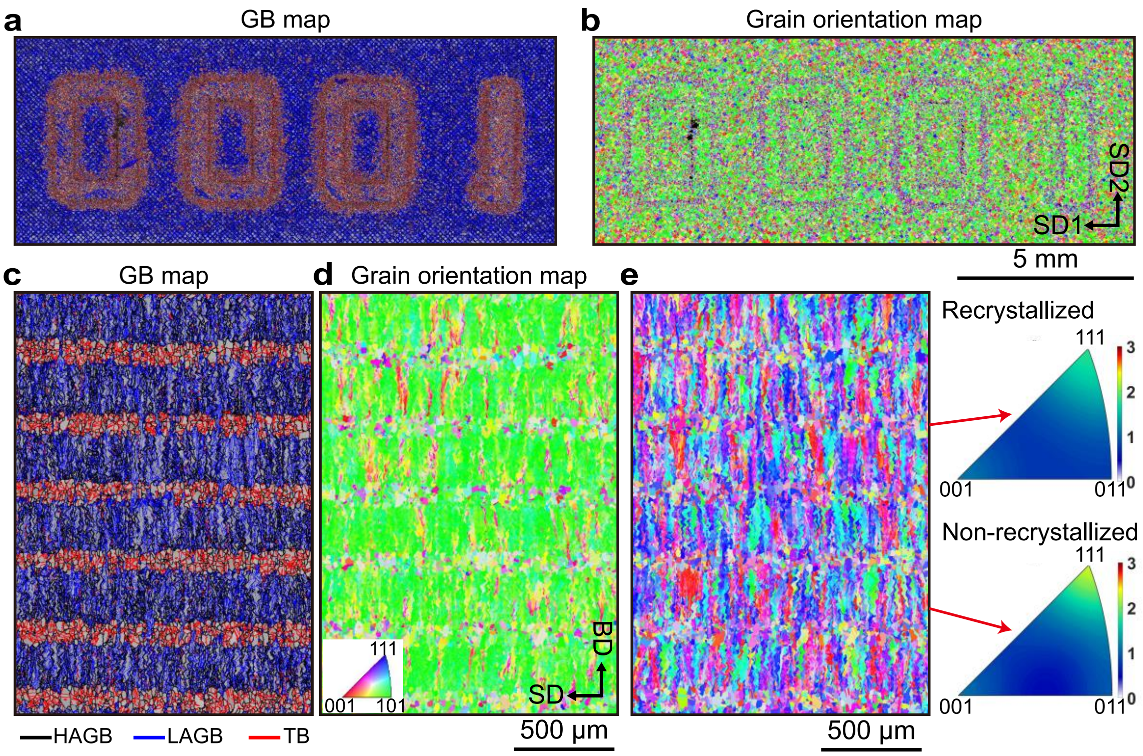

Supplementary Fig. 2. Analysis of the different microstructural features produced through site-specific recrystallization. a Grain boundary (GB) character distribution in the binary code sample as measured by EBSD. **b** Corresponding inverse pole figure map showing crystal orientation within the build plane. **c** Grain boundary character distribution in the layered microstructure sample as measured by EBSD. **d** and **e** Corresponding inverse pole figure maps showing crystal orientation along the build direction and within the build plane, respectively. In (**e**) we show the inverse pole figures from the recrystallized and non-recrystallized layers along tensile direction.

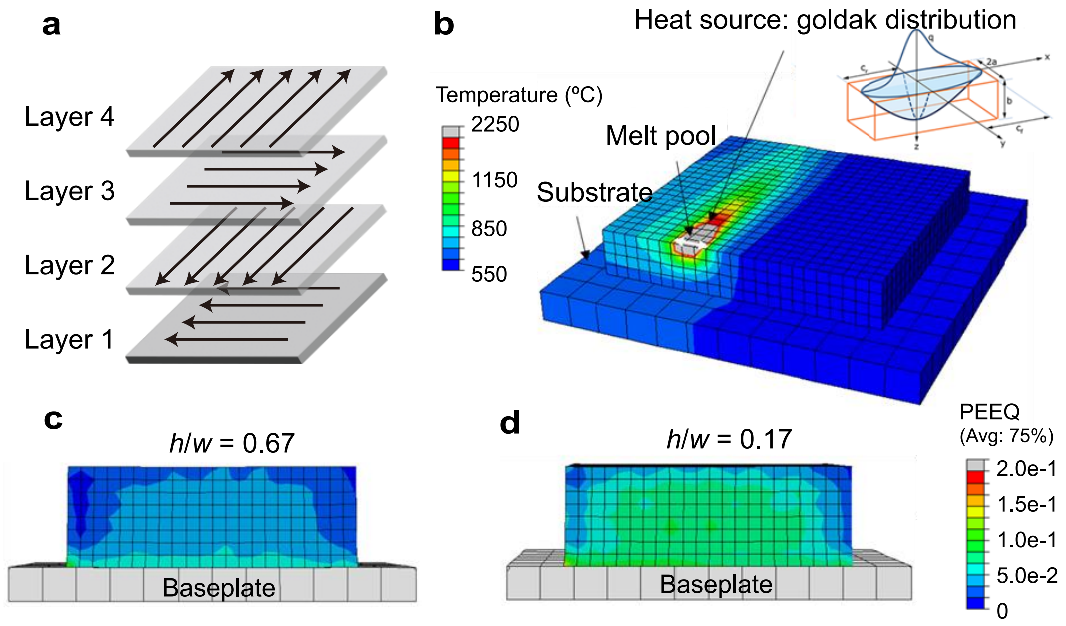

Supplementary Fig. 3. Finite element modelling of the LPBF process. **a** Schematics showing the laser printing strategy employed in the simulation. **b** Temperature field of the SS316L sample in the middle of the build. Regions with temperature higher than the liquidus temperature (1437 ºC) are colored grey and thus represent the melt pool. Distribution of equivalent plastic strains (PEEQ) simulated by FEM as a function of h/w, which are **c** 0.67 and **d** 0.17.

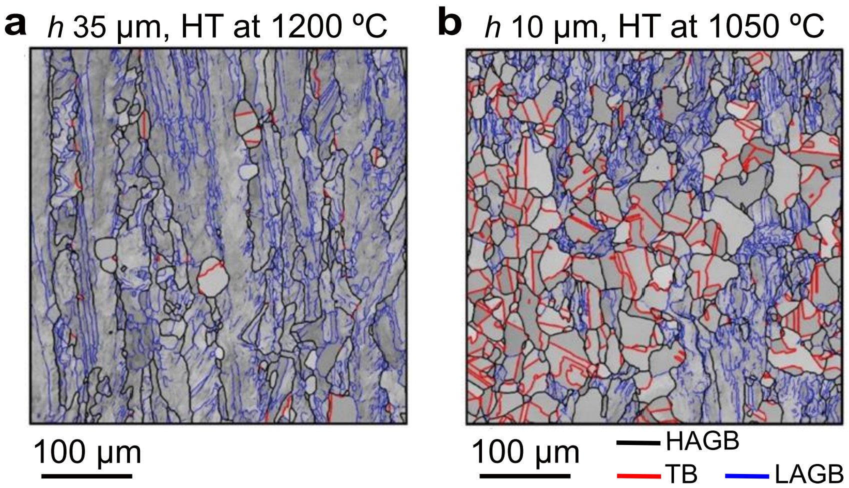

Supplementary Fig. 4. EBSD grain boundary maps showing the thermal stability of SS316L as a function of hatch spacing (*h*). a Produced using *h* = 35 μm and heat treated (HT) at 1200 ºC for 30 mins. **b** Produced using *h* = 10 μm and heat treated at 1050 ºC for 30 mins. Noteworthy is that recrystallized grains nucleate but do not grow in (**a**). HAGB, LAGB, and TB represent high angle grain boundaries, low angle grain boundaries and twin boundaries, respectively.

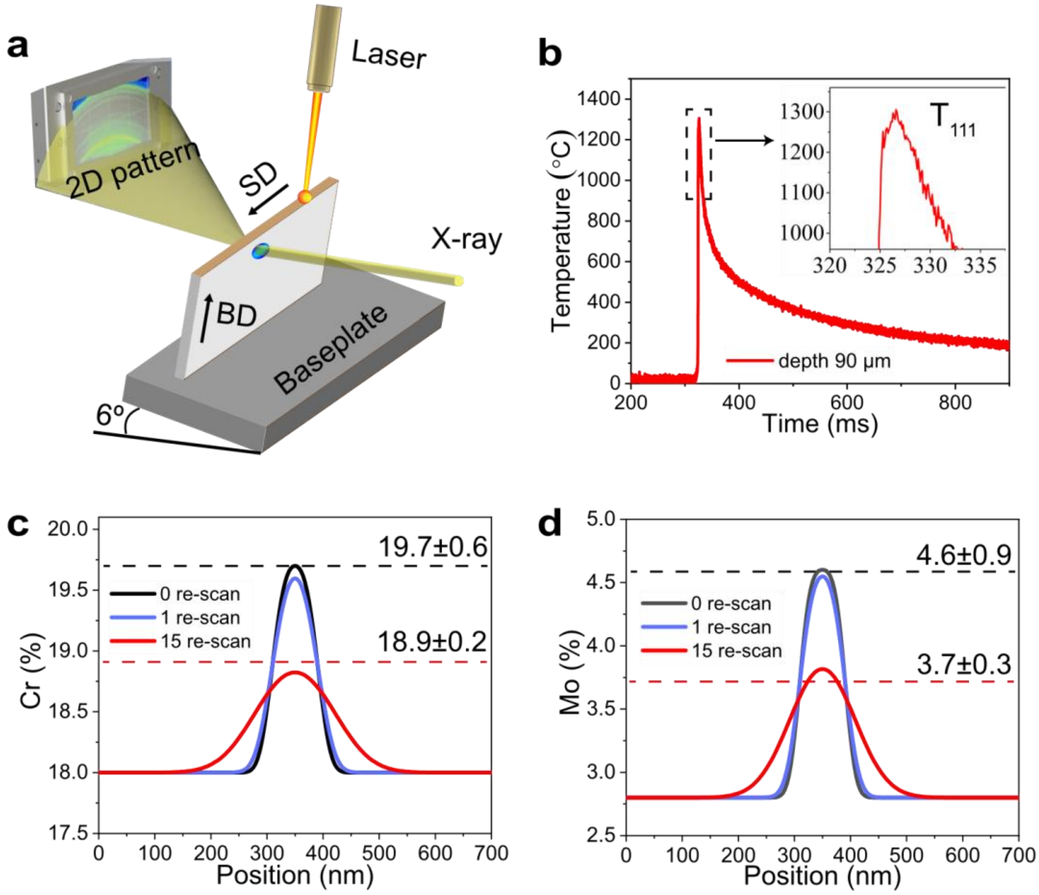

Supplementary Fig. 5. *Operando* X-ray diffraction experiment in transmission mode. a Schematics of *operando* experiment in transmission mode. **b** Temperature profile at a depth of 90 μm derived by tracking the (111) diffraction peak in transmission mode. At this depth, the signal is expected to come from the heat affected zone beneath the melt pool. **c** and **d** DICTRA simulation showing the simulated diffusion of Cr and Mo from a cell boundary into the cell interior as a function of the intrinsic HT produced by laser remelting (i.e., the temperature profile in (**b**)). The dashed lines in (**c**) and (**d**) indicate the element concentrations of cell boundaries measured by STEM-EDS from the top layer, which underwent no remelting, and from a depth of 90 μm, which experienced 15 remelting events, respectively.

The starting profile (0 remelting) is fitted by STEM-EDS analysis of a TEM lamella taken from the top layer of the thin wall. We input the temperature profile obtained from *operando* experiment (Supplementary Fig. 5b) into the DICTRA module of Thermo-Calc to simulate the non-isothermal diffusion driven by one laser

remelting. Upon one laser remelting, the Cr and Mo concentrations at cell boundaries drop from 19.7% and 4.6% to 19.6% and 4.5%, respectively. Supplementary Fig. 5b indicates the depth of the heat affected zone is larger than 90 µm. When producing a 3D build (and not a thin wall), the material may experience intrinsic HTs from multiple layers as well as from adjacent tracks. We remelted the thin wall 15 times and conducted the simulation of solid-state diffusion considering 15 laser remelting events (the red curves in Supplementary Figs. 5c and 5d). We cut a TEM lamella from the same thin wall sample at a depth 90 µm and measured the concentration of Cr and Mo at cell boundaries. The results (Supplementary Figs. 5c and 5d) show a reduction in solute concentration to 18.9% and 3.7%, respectively, which agree very well with the simulations. The combination of *operando* experiments and simulation results prove that the reduction in solute segregation originates from the intrinsic HT generated by multiple laser remelting events.

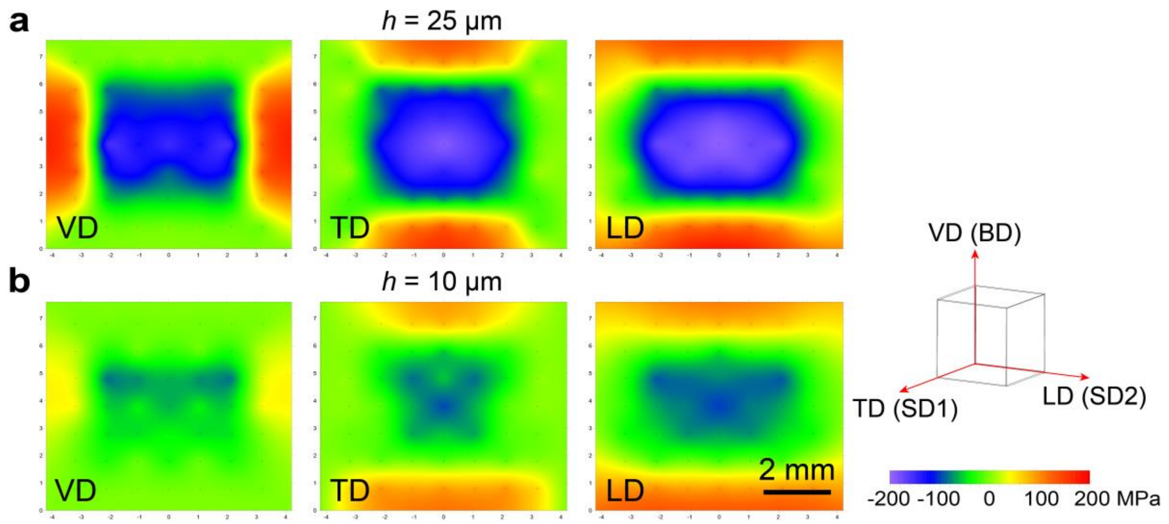

Supplementary Fig. 6. Neutron diffraction characterization of residual stresses in SS316L cubes produced using (a) *h* = 25 μm and (b) *h* = 10 μm. VD, TD, and LD represent vertical, transversal, and longitudinal directions. The stresses are calculated along the three principal directions. The residuals stress evolves from compressive in the core of the sample to tensile near the surface, which is a typical stress distribution in additive manufacturing processes[3, 4].

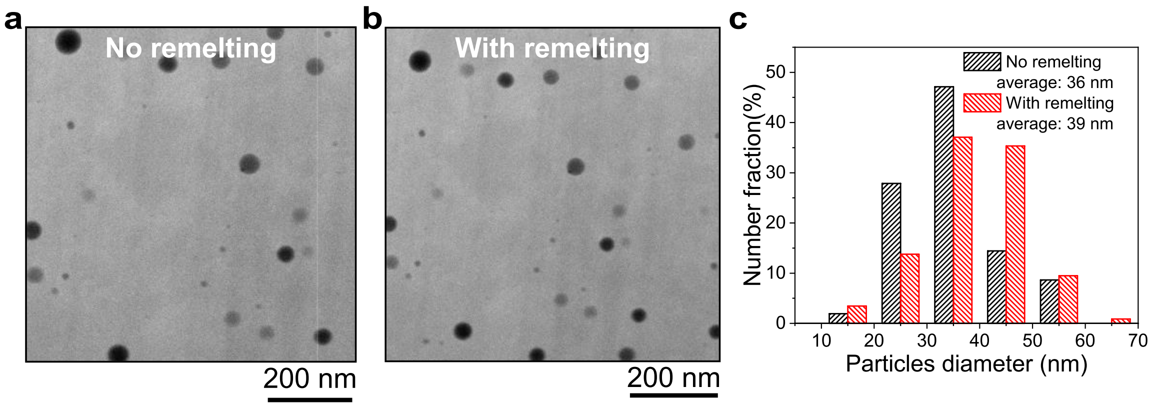

Supplementary Fig. 7. Oxide nanoparticles evolution through the intrinsic heat treatment from laser remelting. High-angle annular dark-field (HAADF) scanning transmission electron microscopy (STEM) images showing the morphology of nanoparticles rich in Si, O, and Mn. (**a**) with and (**b**) without laser remelting. **c** Particle size distribution and corresponding average size (counted over 100 particles). The results show minor particle coarsening in SS316L produced with laser remelting, which likely results from the higher heat input. According to the Zener effect[5], larger particles are more effective at pinning grain boundaries and thus should retard recrystallization. However, our results show that SS316L produced with laser remelting is more prone to undergo recrystallization.

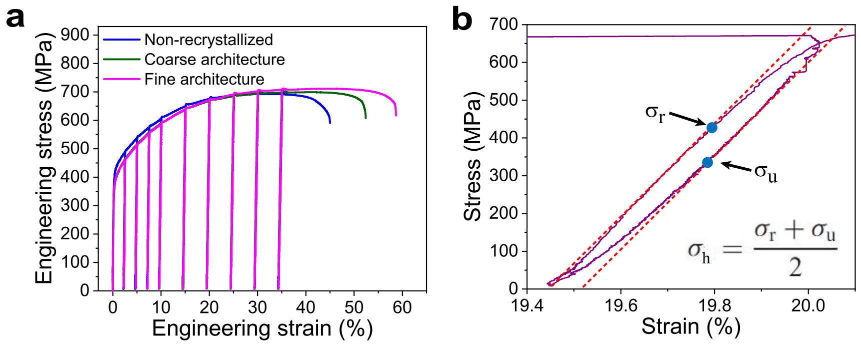

Supplementary Fig. 8. Hetero-deformation induced (HDI) stress analysis. a The unloading–reloading curves for back stress measurements of non-recrystallized microstructure, and the coarse and fine architectures. **b** A measured hysteresis loop from the fine architecture illustrating the method to calculate back stresses at different tensile strains. σ_r represents the yield stress during reloading. σ_u is the stress at the point deviating from the linear behavior upon unloading. A larger hysteresis loop translates into a stronger Bauschinger effect, which is caused by long-range back stresses. Back stress hardening is induced by the pile-ups of directional geometrically necessary dislocations (GNDs), which requires higher applied stress to further deform. The existence of interface zones enhances GND pile-up rate, and thus causes back stresses to increase more rapidly. The equation shown in (**b**) was proposed by Zhu *et al.* and is valid for both back stresses and HDI stress[6].

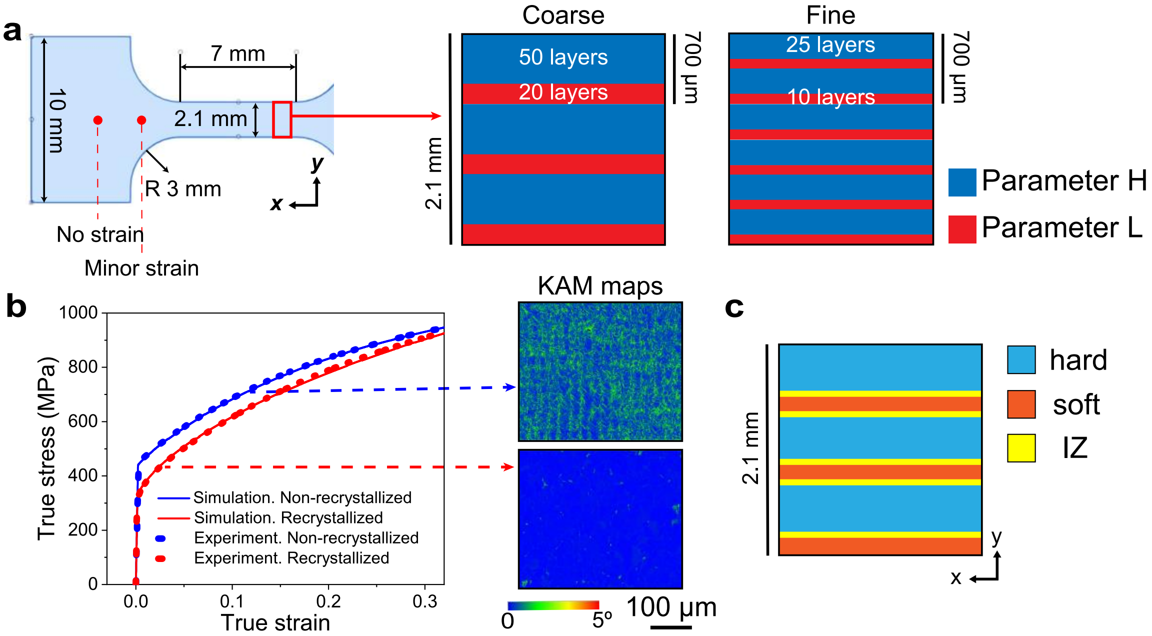

Supplementary Fig. 9. Microstructure architecture design for tensile tests and corresponding simulations. a Geometry of tensile specimens and print design for the coarse and fine architectures. The red dots indicate the approximate locations of the EBSD scan shown in Fig. 5d after tensile test. **b** Calibration of the viscoplastic model on the individual phases (non-recrystallized/recrystallized microstructure) to capture their properties by fitting the experiment tensile test results. **c** Schematic representation of the viscoplastic model containing hard, soft, and a third pseudo-phase at the interface between the other two. This interface zone (IZ) is formed in the soft phase during tensile loading.

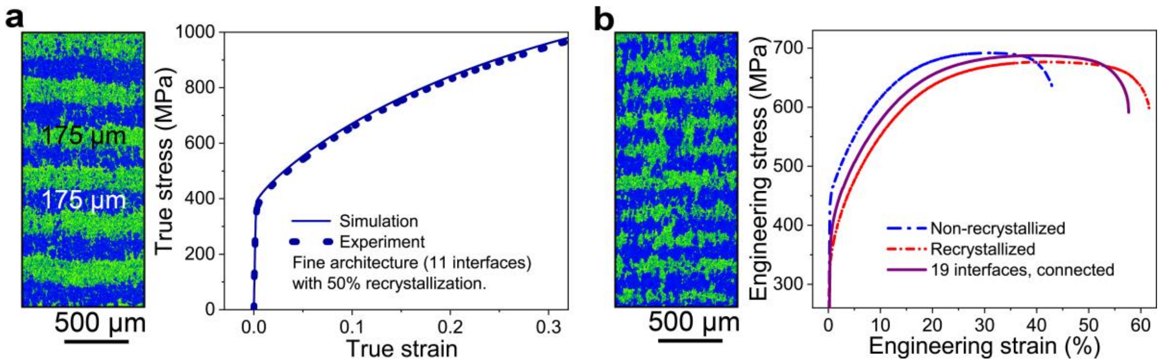

Supplementary Fig. 10. Tensile behavior of other microstructure-layered architectures. a Comparison of experimental and simulated stress-strain curves of a fine architecture with 11 interfaces and a different recrystallization fraction (~50%) compared to that in Fig. 5 in the main text. **b** EBSD map and experimental stress-strain curves of the architecture with 19 interfaces. The interconnected 'soft' microstructure-layers prevent the establishment of large HDI stresses.

Supplementary Reference

1. Waqar S, Guo K, Sun J. FEM analysis of thermal and residual stress profile in selective laser melting of 316L stainless steel. *Journal of Manufacturing Processes* 2021, **66**: 81-100.
2. Kim CS. *Thermophysical properties of stainless steels* (Argonne National Laboratory, United States, 1975).
3. Pant P, Proper S, Luzin V, Sjöström S, Simonsson K, Moverare J*, et al.* Mapping of residual stresses in as-built Inconel 718 fabricated by laser powder bed fusion: A neutron diffraction study of build orientation influence on residual stresses. *Additive Manufacturing* 2020, **36:** 101501.
4. Liu Y, Yang Y, Wang D. A study on the residual stress during selective laser melting (SLM) of metallic powder. *The International Journal of Advanced Manufacturing Technology* 2016, **87**(1)**:** 647-656.
5. Nes E, Ryum N, Hunderi O. On the Zener drag. *Acta Metallurgica* 1985, **33**(1)**:** 11-22.
6. Zhu, Y. & Wu, X. Heterostructured materials. *Progress in Materials Science* **131**, 101019 (2023).
